# Frequent coexistence of anti-topoisomerase I and anti-U1RNP autoantibodies in African American patients associated with mild skin involvement: a retrospective clinical study

**DOI:** 10.1186/ar3334

**Published:** 2011-05-10

**Authors:** Minoru Satoh, Malgorzata E Krzyszczak, Yi Li, Angela Ceribelli, Steven J Ross, Edward KL Chan, Mark S Segal, Michael R Bubb, Eric S Sobel, Westley H Reeves

**Affiliations:** 1 Division of Rheumatology and Clinical Immunology, Department of Medicine, University of Florida, 1600 SW Archer Rd, Gainesville, FL, 32610 USA; 2 Department of Pathology, Immunology, and Laboratory Medicine, University of Florida, 1600 SW Archer Rd, Gainesville, FL, 32610 USA; 3 Department of Oral Biology, College of Dentistry, University of Florida, 1395 Center Drive, Gainesville, FL 32610 USA; 4 Division of Nephrology, Hypertension, and Renal Transplantation, Department of Medicine, University of Florida, 1600 SW Archer Rd, Gainesville, FL, 32610 USA

## Abstract

**Introduction:**

The presence of anti-topoisomerase I (topo I) antibodies is a classic scleroderma (SSc) marker presumably associated with a unique clinical subset. Here the clinical association of anti-topo I was reevaluated in unselected patients seen in a rheumatology clinic setting.

**Methods:**

Sera from the initial visit in a cohort of unselected rheumatology clinic patients (*n *= 1,966, including 434 systemic lupus erythematosus (SLE), 119 SSc, 85 polymyositis/dermatomyositis (PM/DM)) were screened by radioimmunoprecipitation. Anti-topo I-positive sera were also tested with immunofluorescence and RNA immunoprecipitation.

**Results:**

Twenty-five (15 Caucasian, eight African American, two Latin) anti-topo I positive patients were identified, and all except one met the ACR SSc criteria. Coexistence of other SSc autoantibodies was not observed, except for anti-U1RNP in six cases. When anti-topo I alone versus anti-topo I + U1RNP groups were compared, African American (21% vs. 67%), overlap with SLE (0 vs. 50%; *P *= 0.009) or PM/DM (0 vs. 33%; *P *= 0.05) or elevated creatine phosphokinase (CPK) (*P *= 0.07) were more common in the latter group. In comparison of anti-topo I-positive Caucasians versus African Americans, the latter more frequently had anti-U1RNP (13% vs. 50%), mild/no skin changes (14% vs. 63%; *P *= 0.03) and overlap with SLE (0 vs. 38%; *P *= 0.03) and PM/DM (0 vs. 25%; *P *= 0.05).

**Conclusions:**

Anti-topo I detected by immunoprecipitation in unselected rheumatology patients is highly specific for SSc. Anti-topo I coexisting with anti-U1RNP in African American patients is associated with a subset of SLE overlapping with SSc and PM/DM but without apparent sclerodermatous changes.

## Introduction

Autoantibodies to topoisomerase I (topo I, also known as Scl-70) is an established serologic marker of scleroderma (systemic sclerosis, SSc) and associated with diffuse scleroderma and severe interstitial lung disease (ILD) [1-3]. It is highly specific for SSc when tested with standard double immunodiffusion [4,5]; however, several studies using enzyme-linked immunosorbent assay (ELISA) reported high prevalence of anti-topo I in systemic lupus erythematosus (SLE) [6-9], causing confusion and controversies [10,11]. SSc could start from the Raynaud's phenomenon (RP), preceding the onset of SSc for many years, ILD, arthritis, and others [12]. Because autoantibodies are usually produced before typical clinical manifestations, it would not be a surprise to find anti-topo I in undifferentiated connective tissue disease (UCTD), undiagnosed patients [5], or even in certain patients with SLE who are going to develop SSc later [13]. The clinical association of anti-topo I was reevaluated based on radioimmunoprecipitation screening of sera from a cohort of unselected population in a rheumatology clinic that includes undiagnosed patients and patients with a wide variety of diagnoses in addition to established systemic autoimmune rheumatic diseases, such as SSc, SLE, polymyositis/dermatomyositis (PM/DM), and rheumatoid arthritis (RA).

## Materials and methods

### Patients

All 1,966 subjects enrolled in the University of Florida Center for Autoimmune Diseases (UFCAD) registry from 2000 to 2010 were studied. Diagnoses of the patients include 434 SLE, 85 PM/DM, 119 SSc, 35 RA, and 40 Sjögren syndrome (SS). Clinical findings of patients at each visit were evaluated and recorded by the rheumatologists at the Center, following the standard rheumatology clinic evaluation forms of the UFCAD. Diagnoses of patients were by the American College of Rheumatology (ACR) classification criteria for SLE [14,15], SSc [16], and RA [17], the revised European criteria by the American-European Consensus Group for SS [18], and the Bohan's criteria for PM/DM [19]. Mixed connective tissue disease (MCTD) [20] is not classified separately, and SSc patients discussed in this report include patients who also fulfill criteria of other diagnoses (overlap syndrome). ILD was defined by chest radiograph and/or high-resolution computed tomography (HRCT). The protocol was approved by the Institutional Review Board (IRB). This study meets and is in compliance with all ethical standards in medicine, and informed consent was obtained from all patients according to the Declaration of Helsinki.

### Autoantibody analysis

Autoantibodies in sera from the initial visit of each patient were screened by immunoprecipitation (IP) using [^35^S]-methionine-labeled K562 cell extract [21]. RNA components of autoantigens were analyzed with silver staining (Silver Stain Plus; Bio-Rad, Hercules, CA). ACA were examined by immunofluorescence antinuclear antibodies (ANAs) using HEp-2 slides from INOVA Diagnostics (San Diego, CA) and a 1:80-diluted serum.

### Statistical analysis

Prevalence of autoantibodies and clinical manifestation was compared by Fisher Exact test using Prism 5.0 for Macintosh (GraphPad Software, Inc., San Diego, CA). A value of *P *< 0.05 was considered significant.

## Results

### Detection of anti-topoisomerase I and prevalence of anti-topo I in SSc and SLE

**Figure 1:**
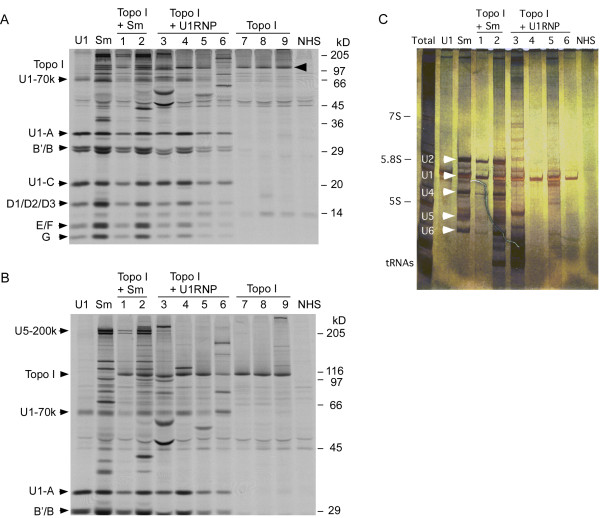
**Coexistence of anti-snRNPs antibodies in anti-topo I-positive sera**. **(a)**12.5% SDS-PAGE. **(b) **8% SDS-PAGE. Six sera with anti-topo I and-snRNPs (two anti-Sm + U1RNP; four anti-U1RNP) were identified by immunoprecipitation of [^35^S]-methionine-labeled K562 cell extract. Positions of Topo I, components of snRNPs (U5RNP-200 kDas; U1-70 kDa; U1-A, B'/B, U1-C, D1/D2/D3, E, F, and G), and molecular weight are indicated. U1, Sm, Topo I, prototype sera for each specificity; Topo I+Sm, anti-topo I with anti-Sm and U1RNP-positive SSc sera; Topo I+U1RNP, anti-topo I and U1RNP-positive SSc sera; NHS, normal human serum. **(c) **Analysis of RNA components in anti-topo I-positive patients with coexisting anti-snRNPs. RNA components immunoprecipitated by human autoimmune sera were analyzed with urea-PAGE and silver staining. Six anti-topo I-positive patients had coexisting anti-UsnRNPs (two anti-Sm (U1, 2, 4 to 6, and 5; lanes 1 and 2) and four anti-U1RNP (lanes 3 to 6)) were identified. Total, total RNAs; U1, Sm, prototype human serum for each specificity; Topo I + Sm, anti-topo I with anti-Sm and U1RNP-positive SSc sera; Topo I + U1RNP, anti-topo I and U1RNP-positive SSc sera; NHS, normal human serum; positions of 7S, 5.8S, and 5S rRNA, tRNAs, and U1, 2, 4, 5, and 6 snRNAs are shown.

Anti-topo I antibodies were detected in 25 (1.3%) of 1,966 subjects enrolled to University of Florida Center for Autoimmune Diseases. Prevalence of anti-topo I in the SSc cohort was 21% (25 of 119); 18% (15 of 85) in Caucasians, 31% (eight of 25) in African Americans, and 25% (two of eight) in Hispanics. An SSc patient of mixed ethnic background did not have anti-topo I. None of the anti-topo I-positive sera had other SSc-specific autoantibodies [3], including anti-RNA polymerase (RNAP) I/III, PM-Scl, or Ku by IP; ACA by immunofluorescence; or anti-U3RNP/fibrillarin or anti-Th/To by RNA analysis from IP. However, six of 25 anti-topo I-positive sera had coexisting anti-U1RNP antibodies, two with anti-Sm. Analysis of protein (Figure 1a, b) and RNA components (Figure 1c) by IP are shown.

Anti-topo I + U1RNP was common in African American (four (16%) of 25) but rare in Caucasian SSc (two (2%) of 85; *P *= 0.02 by the Fisher Exact test). In patients who fulfilled the ACR SLE criteria, anti-topo I was found in three (2%) of 153 in African American, all three cases with anti-U1RNP (two with anti-Sm) and as SLE-SSc overlap syndrome. None of 208 Caucasian or 44 Latin SLE had anti-topo I by IP. Thus, even in unselected patients at our rheumatology clinic, anti-topo I by IP is highly specific for SSc and SSc overlap syndrome.

### Clinical manifestations of patients with anti-topo I versus anti-topo I + U1RNP

Clinical manifestations of 19 patients with anti-topo I versus six patients with anti-topo I + U1RNP were compared (Table 1). All patients fulfilled the ACR SSc classification criteria except for a 48-year-old Caucasian woman with RP, ILD, and polyarthritis. No sclerodermatous changes were noted, and she may be considered systemic sclerosis sine scleroderma. The anti-topo I group was 68% Caucasian, whereas 67% of anti-topo I + U1RNP group was African American (*P *= 0.059). Two of the anti-topo I + U1RNP patients were also positive for anti-Sm (*P *= 0.05; Figure 1). Proximal scleroderma was common (79%) in anti-topo I group. In contrast, three (50%) of six anti-topo I + U1RNP patients had no sclerodermatous skin changes (*P *= 0.03). Overlap with SLE or PM/DM and elevation of creatine phosphokinase (CPK) were common in anti-topo I + U1RNP group (*P *= 0.009 for SLE, *P *= 0.07 for CPK, *P *= 0.05 for PM/DM; Table 1).

**Table 1:** Clinical manifestations of anti-topo I in African American versus Caucasian patients

Specificity	Topo I (*n *= 19)	Topo I + U1RNP (*n *= 6)	*P*
Age (yr, mean ± SD)	55.10 ± 12.9	46.6 ± 8.6	
Male	26%	17%	
Caucasian	68%	33%	
African American	21%	67%	0.059
Latin	11%	0	
Anti-Sm	0	33%	0.05
Proximal scleroderma	79%	50%	
No sclerodermatous changes	5%	50%	0.03
Sclerodactyly only	16%	0	
Pitting scars	74%	83%	
ILD	74%	83%	
Scleroderma kidney	16%	0	
Overlap with SLE	0	50%	0.009
Elevated CPK	11%	50%	0.07
Overlap with PM/DM	0	33%	0.05

CPK, creatine phosphokinase; ILD, interstitial lung disease. *P *values are with the Fisher Exact test.

Clinical features of six cases of anti-topo I with anti-U1RNP are summarized (Table 2). In four African American patients, case 2 had diffuse cutaneous scleroderma (dcSSc) but the other three did not have sclerodermatous skin changes; they fulfilled ACR classification criteria for SSc based on pitting scars and ILD. Overlap of SSc with SLE or PM/DM was seen in three African American cases.

**Table 2:** Clinical characteristic of six cases with anti-topo I coexisting with anti-snRNPs autoantibodies

Case	1	2	3	4	5	6
Anti-snRNPs	Sm, U1RNP	Sm, U1RNP	U1RNP	U1RNP	U1RNP	U1RNP
Race	Afr Am	Afr Am	Afr Am	Afr Am	Caucasian	Caucasian
Type of skin involvement	No scl	dcSSc	No scl	No scl	dcSSc	dcSSc
Pitting scars	Y	Y	Y	Y		Y
ILD	Y	Y	Y	Y	Y	
Raynaud phenomenon	Y	Y	Y	Y	Y	Y
Pulmonary hypertension		Y				
Esophageal dysmotility						Y
Flexion contracture		Y				Y
Acro-osteolysis	Y					P
SLE overlap/number of ACR criteria	Y 6	Y 5	N 2	Y 5	N 2	N 2
PM/DM overlap	Elevated CPK		DM	PM		

Afr Am, African American; dcSSc, diffuse cutaneous scleroderma; F, female; ILD, interstitial lung disease; M, male; Y, present; N, not present; No Scl, no sclerodermatous skin changes; P, possible.

### Racial difference in anti-topo I-positive scleroderma patients

Clinical features of Caucasian versus African American patients with anti-topo I were compared (Table 3). In serology, four (50%) of eight of African Americans with anti-topo I had coexisting anti-U1RNP, two with anti-Sm, but this was only in two (13%) of 15 Caucasians. Proximal scleroderma was noted in 87% of Caucasians but only in 38% of African Americans (*P *= 0.03). Three of eight African American anti-topo I-positive patients did not have sclerodermatous changes, and two had sclerodactyly only (*P *= 0.03, no skin changes and sclerodactyly only combined). Overlap with SLE and elevated CPK (*P *= 0.03 versus Caucasians) and overlap with PM/DM (*p *= 0.05) were also common in African Americans.

**Table 3:** Clinical manifestations of African American versus Caucasian patients with anti-topo I

	Caucasian (*n *= 15)	African American (*n *= 8)	*P*
Age (yr, mean ± SD)	56.5 ± 11.5	45.9 ± 13.2	
Male	20%	38%	
Anti-U1RNP	13%	50%	0.13
Anti-Sm	0	25%	
Proximal scleroderma	87%	38%	0.03
No skin changes	7%	38%	0.03^a^
Sclerodactyly only	7%	25%	
Pitting scar	80%	88%	
ILD	73%	75%	
Scleroderma kidney	20%	0	
Overlap with SLE	0	38%	0.03
Elevated CPK	7%	50%	0.03
Overlap with PM/DM	0	25%	0.05

^a^No skin changes and sclerodactyly combined. CPK, creatine phosphokinase; ILD, interstitial lung disease. *P *values are with the Fisher Exact test.

Lack of skin changes, and overlap with SLE and PM/DM are common in African American patients with anti-topo I + U1RNP but not anti-topo I antibodies alone. These clinical features were not present in two cases of anti-topo I + U1RNP in Caucasians, suggesting that this clinical subset may be relatively unique to African Americans.

## Discussion

Anti-topo I is a highly specific disease marker of SSc when tested by immunodiffusion [4,5] or IP as in the present study. It can be occasionally found in undiagnosed patients such as UCTD [22] or RP [5], at least partially, because autoantibodies are usually produced before clinical manifestation [23]. In one study, anti-topo I were tested by ELISA in 2,181 unselected individuals to find none was positive [24]. All these data support the high specificity of anti-topo I for SSc.

Reports on high prevalence of anti-topo I in SLE by ELISA and its association with SLE activity and nephritis [8,9] challenged the general observation on SSc specificity of anti-topo I and triggered much confusion and many controversies [5,10,11]. When we tested 46 SLE sera (from Louisiana, not included in the present study) by a commercial anti-topo I ELISA, 41% were positive; however, only two of 19 were IP positive [10]. In the study that had 32 (25%) of 128 prevalence of anti-topo I in SLE [8], only four of 32 ELISA positives were double immunodiffusion positive, and data supporting the specificity of ELISA were limited. Some also reported 13% to 29% prevalence of anti-topo I in SLE [6,7,9,25] whereas others reported low prevalence by ELISA [5,11]. Thus, the prevalence of anti-topo I in SLE appears to depend on the source of antigens or ELISA kits. In some studies [8-10], anti-topo I ELISA positives in SLE are detecting antibodies that are different from those detected by immunodiffusion and IP. False positives caused by anti-dsDNA/chromatin antibodies in SLE sera in ELISA for autoantibodies to DNA-binding proteins, such as Ku and replication protein A, are well documented [10,26]. Thus, the most likely explanation appears to be that anti-topo I ELISA positives in SLE are false positives caused by antibodies to DNA/chromatin. Because topo I is a nucleotide sequence nonspecific DNA-binding protein, one scenario is that serum DNA binds to topo I coated on plate, and this is followed by anti-DNA/chromatin antibodies binding to DNA. A second scenario is that preformed serum anti-DNA/chromatin immune complex can bind to topo I via its DNA component. It is also possible that anti-topo I ELISA positives in SLE in some studies reflect detection of low-affinity antibodies or antibodies other than IgG class because of secondary antibody specificity. Alternatively, certain ELISA antigens may contain impurities as unrelated antigens, or some SLE sera recognize denatured topo I epitopes not present in native molecules and thus appear unreactive (negative) in immunodiffusion or IP.

Anti-topo I antibodies are positive in 1% to 3% of SLE patients, even by reliable methods such as immunodiffusion [8]. This may be explained by SLE-SSc overlap syndrome, not typical pure SLE [10,27], as shown in the present study. Thus, anti-topo I by immunodiffusion or IP is specific for SSc, and cautious interpretation is required for anti-topo I ELISA positive results in SLE.

SSc patients can be classified based on autoantibody specificities that are associated with unique clinical subsets [3]. Coexistence of SSc-related autoantibodies is uncommon [3]; however, a combination of anti-topo I and anti-U1RNP appears to be an interesting and possibly clinically useful exception. In addition to cases reported mainly from Japan [27-29], frequent association of anti-topo I and anti-U1RNP in a large Japanese and American cohorts also was observed [1,2]. In one study, nine (12%) of 78 of anti-topo I-positive SSc had coexisting anti-U1RNP, and an additional three later developed anti-U1RNP [1]. Three patients in this cohort also had anti-Sm antibodies [27]. A study from Finland reported 12% of coexistence of anti-topo I and anti-U1RNP [30]. Detection of anti-topo I in MCTD patients indicates coexisting anti-topo I and anti-U1RNP [31]. Regarding the issue of race and coexistence of these two specificities in SSc, the prevalence was reported as 2% in Caucasian, 13% in African American, and 16% in Japanese in another U.S. cohort [2]. The 50% prevalence of anti-U1RNP in anti-topo I-positive African Americans in the present study is higher than that in other studies to date. Furthermore, prevalence of diffuse scleroderma in African Americans was low versus that in the previous study [2]. Three of four cases of anti-topo I + U1RNP-positive African American patients can be classified as SSc by using the ACR criteria based on the presence of pitting scars and ILD [16]; however, they lack sclerodermatous skin changes. Thus, this subset of patients might not be included in the studies that selected SSc patients based on diagnosis by physicians [2,32,33], sclerodactyly as a minimum requirement [34], or by using other SSc criteria [35]. They can be easily classified as "SLE with ILD and RP" because this is the common pattern of presentation among anti-U1RNP-positive SLE or MCTD. This subset could also be real anti-topo I-positive SLE without features of SSc described in some literature [8]. It may be clinically important to identify anti-topo I, in addition to anti-U1RNP, in these patients, because the former could be associated with severe ILD and scleroderma renal crisis [2,3].

## Conclusions

Anti-topo I detected by IP in unselected rheumatology patients is highly specific for SSc. Anti-topo I and anti-U1RNP frequently coexist in African American patients, and they are associated with a subset of overlap syndrome of SLE, SSc, and PM/DM, characterized by RP, pitting scars, and ILD without sclerodermatous changes.

## Abbreviations

ACR: American College of Rheumatology; ANA: antinuclear antibodies; CPK: creatine phosphokinase; dcSSc: diffuse cutaneous scleroderma; HRCT: high-resolution computed tomography; ILD: interstitial lung disease; IP: immunoprecipitation; IRB: Institutional Review Board; MCTD: mixed connective tissue disease; PM/DM: polymyositis/dermatomyositis; RA: rheumatoid arthritis; RNAP: RNA polymerase; RP: Raynaud's phenomenon; SLE: systemic lupus erythematosus; SS: Sjögren syndrome; SSc: systemic sclerosis: scleroderma; Topo I: topoisomerase I; UCTD: undifferentiated connective tissue disease; UFCAD: University of Florida Center for Autoimmune Diseases.

## Competing interests

The authors declare that they have no competing interests.

## Authors' contributions

MS, MEK, YL, SJR, and EKLC carried out the immunoassays, and MS designed the study and performed the statistical analysis. MSS, MRB, ESS, and WHR enrolled patients for the study and maintained the database. MS, AC, and EKLC drafted the manuscript. All authors read and approved the final manuscript.
